# Structure–Activity Relationships and Therapeutic Applications of Retinoids in View of Potential Benefits from Drug Repurposing Process

**DOI:** 10.3390/biomedicines12051059

**Published:** 2024-05-10

**Authors:** Piotr Kawczak, Igor Feszak, Piotr Brzeziński, Tomasz Bączek

**Affiliations:** 1 Department of Pharmaceutical Chemistry, Faculty of Pharmacy, Medical University of Gdańsk, 80-416 Gdańsk, Poland; tomasz.baczek@gumed.edu.pl; 2 Department of Nursing, Faculty of Health Sciences, Pomeranian University in Słupsk, 76-200 Słupsk, Poland; igorfeszak@gmail.com; 3 Department of Physiotherapy and Medical Emergency, Institute of Health Sciences, Pomeranian University in Słupsk, 76-200 Słupsk, Poland; piotr.brzezinski@upsl.edu.pl; 4 Department of Dermatology, Voivodeship Specialist Hospital, 76-200 Słupsk, Poland

**Keywords:** retinoids, structure–activity, therapeutic applications, drug repurposing

## Abstract

Vitamin A, an essential micronutrient, is integral to various biological processes crucial for organismal development and maintenance. Dietary sources of vitamin A encompass preformed retinol, retinyl esters, and provitamin A carotenoids. Retinoic acid (RA), a key component, plays pivotal roles in vision, cell proliferation, apoptosis, immune function, and gene regulation. Drug repurposing, an effective strategy for identifying new therapeutic applications for existing drugs, has gained prominence in recent years. This review seeks to provide a comprehensive overview of the current research landscape surrounding retinoids and drug repurposing. The scope of this review encompasses a comprehensive examination of retinoids and their potential for repurposing in various therapeutic contexts. Despite their efficacy in treating dermatological conditions, concerns about toxicity persist, driving the search for safer and more potent retinoids. The molecular mechanisms underlying retinoid activity involve binding to retinoic acid receptors (RARs) and retinoid X receptors (RXRs), leading to transcriptional regulation of target genes. This review seeks to shed light on the possibilities for repurposing retinoids to cover a wider spectrum of therapeutic uses by exploring recent scientific progress. It also aims to offer a more comprehensive understanding of the therapeutic prospects of retinoids and the broader impact of drug repositioning in contemporary medicine.

## 1. Introduction

Retinoids comprise a diverse class of chemical compounds derived from vitamin A. Their impact on biological systems is extensive, largely due to their interaction with specific nuclear receptors. Retinoic acid (RA), a prominent derivative of vitamin A, exerts significant influence over various biological processes. It is crucial for maintaining vision, regulating cell growth and differentiation, controlling programmed cell death (apoptosis), and orchestrating embryonic development [1,2]. Beyond these functions, RA promotes the formation of bone tissue, supports key immune system activities, and contributes to the suppression of tumorigenesis by activating specific tumor-suppressor genes. The complexity and versatility of retinoid activity have attracted considerable attention from the scientific and medical communities, particularly in the context of drug repurposing. Vitamin A, an essential micronutrient, plays a pivotal role in a multitude of biological functions that are fundamental for the proper development and maintenance of living organisms [3,4]. It is involved in critical processes such as tissue differentiation, cellular growth, mucus secretion, embryonic development, and cellular homeostasis. Humans obtain vitamin A through dietary sources, including preformed retinol from animal products, retinyl esters from fortified foods, and provitamin A carotenoids from a variety of fruits and vegetables [1,3,5]. This review aims to provide an in-depth exploration of retinoids and their emerging role in drug repurposing, with a specific focus on their potential to be redirected towards new therapeutic applications.

Drug repurposing, sometimes called drug repositioning, is the practice of investigating fresh medical uses for drugs that are already on the market. This approach is effective for uncovering or creating drug molecules with novel pharmacological or therapeutic uses [6,7,8]. In recent times, numerous pharmaceutical companies have embraced the drug repositioning strategy to discover new drugs by identifying unique biological targets in their drug development programs [8,9,10]. This method proves highly efficient, saving time and costs, while minimizing the risk of failure. By maximizing the therapeutic potential of a drug, it significantly enhances the success rate. Consequently, drug repositioning emerges as a viable alternative to the traditional drug discovery process. The conventional method of discovering new molecular entities (NME) through traditional or de novo approaches is known for its lengthy, time-consuming, and expensive nature [10,11,12]. In contrast, drug repositioning combines activity-based experimental approaches with in silico computational methods to rationally identify new uses for existing drugs. This emerging strategy leverages the safety-tested nature of drugs in humans, redirecting them based on a valid target molecule. This targeted redirection is particularly valuable in addressing rare, difficult-to-treat diseases, as well as neglected diseases [13,14,15].

The scope of this review encompasses a comprehensive examination of retinoids and their potential for repurposing in various therapeutic contexts. This includes exploring the molecular mechanisms that govern retinoid activity, focusing on the role of retinoic acid receptors (RARs) and retinoid X receptors (RXRs) in gene regulation [16,17,18]. These receptors mediate the effects of retinoids, playing a critical role in modulating a range of biological processes. Understanding the interaction between retinoids and these receptors is crucial for identifying new therapeutic avenues [18,19,20].

One of the most significant applications of drug repurposing with retinoids is in oncology [21,22,23]. Specific retinoid compounds have shown promising results in the treatment of certain cancers, such as acute promyelocytic leukemia (APL) [23,24,25]. Additionally, there is growing evidence supporting the use of retinoids in modulating immune responses, which could open new pathways for treating autoimmune diseases and other chronic conditions [24,25,26]. However, the repurposing of retinoids is not without challenges. Issues such as optimal dosing, adverse side effects, and patient compliance must be carefully managed to ensure successful outcomes [27,28,29]. Despite these obstacles, drug repositioning represents a valuable opportunity for addressing unmet medical needs, particularly for rare and neglected diseases [28,29,30].

Through an examination of scientific advancements, this review aims to illuminate the potential of retinoids to be repurposed for a broader range of therapeutic applications together with offering a deeper understanding of the therapeutic potential of retinoids and the broader implications of drug repositioning in modern therapeutics.

## 2. Structure–Activity of Natural and Synthetic Retinoids

The retinoids, comprising over 4000 natural and synthetic molecules, are closely linked to all-trans retinoic acid (ATRA), a metabolite of vitamin A (retinol). ATRA, a crucial regulator in chordate embryogenesis and adult homeostasis, influences processes such as embryonic development, vision, cellular differentiation, proliferation, and apoptosis [31,32,33]. Despite being effective in treating specific dermatological conditions, retinoids hold promise as chemopreventative and chemotherapeutic agents. However, concerns about toxicity have limited their broader application [34,35,36].

**Figure 1:**
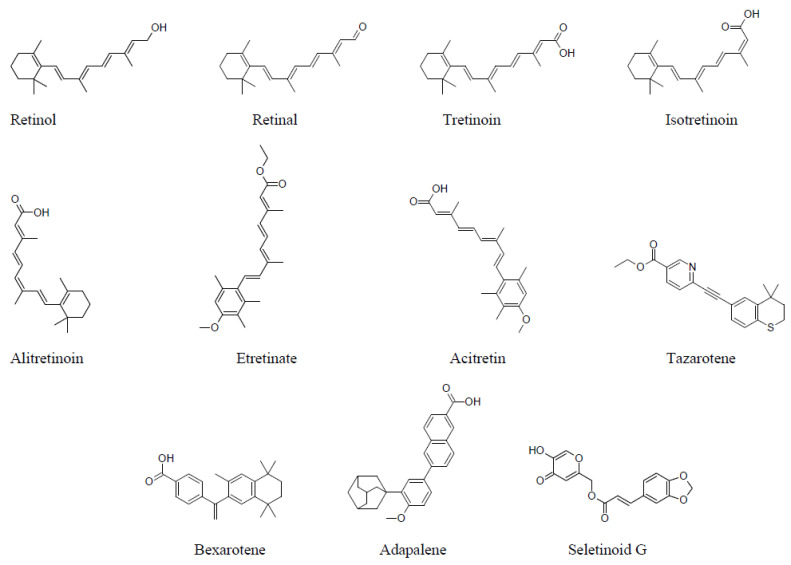
The generations of retinoids.

Three generations of retinoids have been defined, each with distinct compounds: first-generation includes retinol, retinal, tretinoin (all-*trans* retinoic acid; ATRA), isotretinoin (13-*cis*-retinoic acid; 13cRA), and alitretinoin (9-*cis*-retinoic acid; 9cRA); second-generation includes etretinate and acitretin; and third-generation includes tazarotene, bexarotene, and adapalene. By incorporating aromatic rings into their molecular structures for enhanced rigidity (unlike the more flexible aliphatic backbone found in vitamin A and older retinoids) these compounds reduce the potential for off-target effects. A fourth generation, represented by seletinoid G, introduces a pyranone-derived retinoid, and is effective for treating intrinsic and photo-aging because it does not irritate the skin when applied topically under occlusion, whereas tretinoin tends to cause intense redness [1,31]. All the generations’ structural formulas are represented in Figure 1. Despite advancements, the quest for potent retinoids with enhanced efficacy and reduced toxicity continues, especially in the realms of dermatology and oncology [37,38]. The evolving landscape of retinoid development underscores the ongoing efforts to balance therapeutic benefits with safety considerations.

The existence of five linked double bonds causes ATRA to absorb light in the visible and near-UV spectrum, making it unstable when exposed to normal lab lighting. The different isomers of ATRA interact with different receptors, triggering unique biological responses [31]. To address this issue, the development of stable synthetic analogues with a suitable design can overcome the challenges associated with the photoinstability of ATRA [39,40].

Animals cannot produce Vitamin A (retinol), so it must come from dietary sources. This can be achieved either by consuming provitamin A carotenoids found in plants, such as β-carotene, or by eating animal-based foods containing vitamin A. Once ingested, vitamin A is stored as retinyl esters in the liver until it is needed. When required, it is transported in the blood bound to retinol-binding protein (RBP) [41,42,43]. Retinol can be converted back and forth to retinal through oxidation by retinol dehydrogenases (RDH), and retinal can be further oxidized to ATRA by retinaldehyde dehydrogenases (RALDH) or cytochrome P450 enzymes in the liver [43,44,45].

ATRA can cross the plasma membrane without the need for active transport, and it is carried to the cell nucleus by cellular retinoic acid-binding proteins (CRABP-I and II), where it attaches to nuclear receptors. On the other hand, when unbound, ATRA can be stored by binding to retinoic acid-binding protein I (CRABP-I), which prevents it from exerting its biological effects [31]. It may also undergo oxidation by cytochrome P450 enzymes in collaboration with CRABP-I, resulting in the formation of polar metabolites such as 4-hydroxy-retinoic acid and 18-hydroxy-retinoic acid [45,46,47].

Retinoids primarily exert their effects by regulating the transcription of certain genes, a process that involves their binding to receptors within the steroid/thyroid superfamily of nuclear receptors. There are two types of retinoid nuclear receptors: retinoic acid receptors (RARs) and retinoid X receptors (RXRs). Each class has unique structural and functional characteristics. In both cases, these nuclear receptors act as ligand-dependent transcriptional regulators, utilizing activation functions (AFs) to influence gene expression. [32,48].

**Figure 2:**
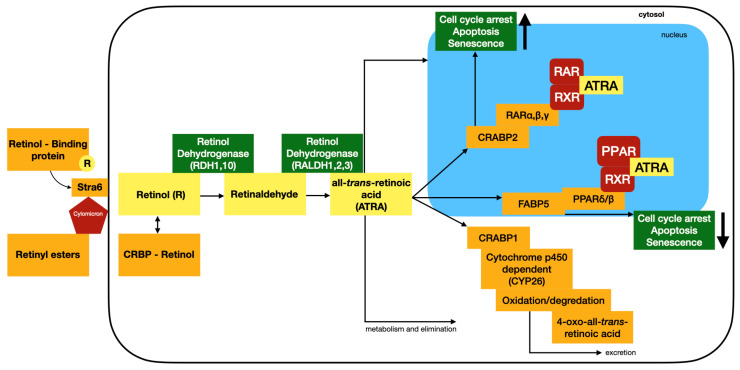
Cellular retinoic acid signaling, based on Ref. [1]: cellular retinoic-acid-binding proteins (CRABPs), cellular retinol-binding proteins (CRBPs), fatty-acid-binding protein (FABP), peroxisome proliferator-activated receptor (PPAR), retinaldehyde dehydrogenases (RALDH), retinol dehydrogenases (RDH), and membrane receptor stimulated by RA 6, which mediates cellular uptake of vitamin A (STRA6).

Natural retinoids like ATRA and 9cRA have limited effectiveness because they can undergo isomerization, producing variants with different activities, and they can be broken down by cytochrome P450 enzymes. These degrading processes affect key functional groups in ATRA and 9cRA. To address this issue, replacing these vulnerable moieties with more robust pharmacophores allows for the synthesis of retinoids with similar efficacy as ligands for RARs or RXRs. Importantly, these modified retinoids exhibit enhanced resistance to isomerization and metabolism, leading to improved overall activities [31]. Cellular retinoic acid signaling is presented in Figure 2.

Synthetic retinoids containing one or more aromatic rings are referred to as arotinoids. Through these modifications, it becomes possible to replace the trimethylcyclohexenyl ring and the conjugated tetraene present in natural retinoids with more durable structural units such as stilbene, tolan, or biaryl. This substitution results in the synthesis of retinoids that not only demonstrate high activity but also exhibit enhanced stability [31].

The effect of altering the hydrophobic segment on retinoid activity can be studied by examining structure–activity relationships in a series of arotinoid acid (TTNPB) analogues. Arotinoid acid is a retinoid that consists of benzoic acid substituted at position four by a 2-(5,5,8,8-tetramethyl-5,6,7,8-tetrahydronaphthalen-2-yl)prop-1-en-1-yl group, and is a synthetic retinoid that acts as a selective agonist for the RAR. These relationships are studied in the context of the differentiation of cultured hamster trachea cells using the TOC assay (Tracheal Organ Culture assay). The assay serves as a method to investigate and understand how alterations in the hydrophobic component of retinoids influence their activity, particularly in the differentiation process of cultured hamster trachea cells [49,50,51].

**Figure 3:**
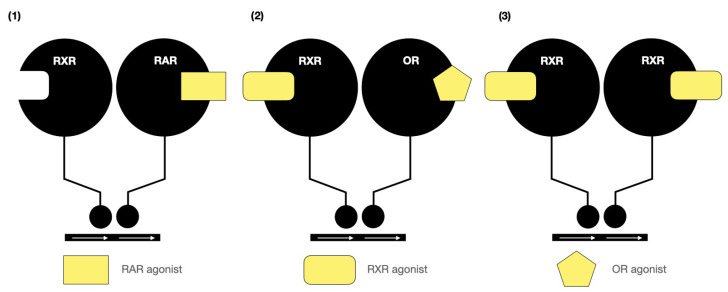
Multiple retinoid signaling pathways, based on Ref. [31]: retinoic acid receptor (RAR), retinoid X receptor (RXR), and other receptor (OR).

To bind effectively to the ligand-binding pockets (LBPs) of the retinoic acid receptor (RAR) or the retinoid X receptor (RXR), the polar end of the retinoid must be able to form favorable interactions with the amino acid residues at the “bottom” of the LBP. These specific interactions, involving the LBP and the “anchoring” group at the polar end, are visible in the crystal structures of 9cRA with both RAR and RXR. This insight into the molecular interactions sheds light on the mechanisms governing the binding affinity and specificity of retinoids to their respective receptors [31,52]. Multiple retinoid signaling pathways are presented in Figure 3.

Many arotinoids share a common structural motif, featuring 1,1,4,4-tetramethyl-1,2,3,4-tetrahydronaphthalene as the hydrophobic unit and a carboxylate-bearing aromatic ring as the polar terminus. These two functionalities are connected by a short linker unit typically consisting of 1–3 atoms. Despite their small size, a wide array of functionalities has been employed as linkers in arotinoid structures. The variations in linker structure allow for the control of selectivity between RARs and retinoid X receptors (RXRs), as well as among different RAR isotypes. This structural flexibility contributes to the fine-tuning of arotinoid properties and their interactions with specific receptors [31].

It has been found that retinoids with large, bulky groups like p-tolyl or biphenyl on their hydrophobic section can disrupt the proper alignment of helix H12 in the ligand-binding domain (LBD). This misalignment stabilizes the receptor-co-repressor complex, causing a suppression of transcription. Retinoids with these features are called inverse agonists or negative antagonists because they inhibit the receptor’s transcriptional activity by encouraging the formation of a repressive complex [53,54].

Certain synthetic retinoids interfere with the receptor’s interaction with co-repressors without creating a surface on the ligand-binding domain (LBD) of the retinoic acid receptor (RAR) where co-activators can attach. In the absence of retinoid X receptor (RXR) agonists, this disruption keeps transcription at basal levels. These retinoids are known as neutral antagonists. What’s interesting is that even after a neutral antagonist binds to the RAR, co-activators can still be recruited to the RAR/RXR heterodimer when RXR agonists are present, leading to transcription activation similar to what is seen with RAR agonists. Neutral antagonists of RARs typically have slightly smaller “bulky groups” on their hydrophobic part compared to analogous inverse agonists [55].

In mammals, three distinct types of retinoic acid receptors (RARs) are encoded by separate genes: RARα, RARβ, and RARγ. These types differ in their amino acid sequences, particularly at three key points within their ligand-binding pockets (LBPs), located in the H3, H5, and H11 helices, respectively (Table 1). Furthermore, each type of RAR has multiple variants, known as isoforms, such as RARα1 and RARα2, RARβ1 through RARβ4, and RARγ1 and RARγ2. The isoforms within a given type vary only in their A regions [56].

Moreover, there are three mammalian retinoid X receptor (RXR) isotypes: RXRα, RXRβ, and RXRγ. These isotypes also exhibit differing amino acid sequences, although the residues in their LBPs are identical. Each RXR isotype has two known isoforms: RXRα1 and RXRα2, RXRβ1 and RXRβ2, and RXRγ1 and RXRγ2. This diversity in RAR and RXR isotypes and isoforms contributes to the complexity of retinoid signaling pathways in mammals [57].

The ligand-binding pocket (LBP) of RARα differs from that of the β and γ isotypes due to the presence of a hydrogen-bond donor residue, Ser232, on helix H3. This is in contrast to the lipophilic Ala225 and Ala234 residues found in the LBPs of RARβ and RARγ, respectively. In RARβ, the LBP lacks the hydrogen-bond donor residue Ser232, preventing selective interaction with ligands through hydrogen bonding. Instead, RARβ has smaller Ala225 and Ile263 residues in the hydrophobic region of the LBP, resulting in a larger binding cavity [31].

Similarly, the LBP of RARγ also lacks the hydrogen-bond donor residue Ser232, found in RARα, and features smaller Ala225 and Ile263 residues, like RARβ. Additionally, the RARγ LBP differs from RARα and RARβ by the presence of the weakly polar Met272 residue. The formation of a weak hydrogen bond between this specific residue and retinoids that have a hydrogen-bond donor located on or near the hydrophobic region confers selectivity for RARγ. These structural differences contribute to the distinct ligand-binding properties and selectivities of RARα, RARβ, and RARγ isotypes [31].

Synthetic retinoids offer several benefits compared to their natural counterparts, like ATRA and 9cRA. In addition to generally being more resistant to light and enzymatic breakdown, which enhances their activity, RAR isotype-specific retinoids provide more flexibility in managing their effects. This is especially useful in medical settings as it helps reduce toxicity. Additionally, selective retinoids allow researchers to delve into particular signaling pathways driven by different retinoid receptors, with a focus on those in the nervous system. This targeted approach allows for a more precise understanding and modulation of retinoid-related mechanisms, paving the way for potential therapeutic applications and advancements in various medical fields.

## 3. Therapeutic Applications and Drug Repurposing

The FDA has approved the use of retinoids to treat the following conditions: acne vulgaris—isotretinoin, psoriasis—acitretin, and cutaneous T-cell lymphoma—bexarotene.

The pivotal role of retinoids is in treating severe forms of acne, particularly when other therapeutic methods fail. However, their clinical applications extend to other dermatological conditions, going beyond acne, which underscores their versatility as a therapeutic agent in dermatology. In this chapter, we will discuss in detail the varied clinical uses of retinoids, focusing primarily on their most common representative, isotretinoin (ISO). We will try to provide numerical data and study results to illustrate their efficacy and therapeutic potential.

ISO is best known for its unmatched efficacy in treating severe forms of common acne (e.g., acne vulgaris). Acne, especially in its cystic form, can be resistant to standard treatment methods, including antibiotics and topical medications. In such cases, ISO is often the drug of choice. Studies have shown that ISO offers complete remission in a significant majority of patients after just one therapeutic session. The first such study appeared in 1979 and reported complete remission in 13 of 14 patients. Since the first guidelines on safe and optimal doses of ISO were introduced in 1992, an increasing number of randomized clinical trials and meta-analyses have been published, declaring the high efficacy of ISO in inducing long-term remissions. One of the larger prospective studies on a group of 305 patients with acne vulgaris, who used ISO at an optimal dose of 0.5–1.0 mg/kg (cumulative max dose of 120 mg/kg) for 16–32 weeks, reported remission in 87.64% of patients, with only 16.39% experiencing a relapse during a 6-month follow-up [58,59,60,61].

Although retinoids are primarily used in the treatment of acne vulgaris, they have also been found effective in treating rosacea. Rosacea is a chronic skin disease characterized by facial redness, papules, and pustules. ISO in low doses, has shown effectiveness in reducing the symptoms of rosacea, especially in cases resistant to other forms of treatment. Studies have demonstrated that ISO is effective in treating rosacea with papulopustular symptoms, leading to significant improvement in the majority of patients [62,63].

Among retinoids, ISO and acitretin are the most commonly used in the treatment of Hidradenitis suppurativa (HS), although their efficacy and mechanisms of action vary depending on individual cases. HS is a chronic, inflammatory skin condition characterized by the presence of painful nodules, abscesses, and fistulas, primarily in the armpits, groin, and under the breasts. The etiology of HS is complex, including disorders within the hair follicles, excessive bacterial colonization, and inflammatory states. Treating HS is challenging and often requires a multidisciplinary approach. The evidence regarding the effectiveness of retinoids in HS is mixed and largely based on case studies and small case series. In one of the larger retrospective studies on HS, the majority of participants, 68%, showed a partial or complete response to ISO treatment. Beyond pharmacological and conservative treatments, which are often insufficient, HS ultimately requires surgical intervention to maintain consistent remission of lesions [64,65].

Retinoids are also used in the treatment of ichthyosis (fish scale disease) and other keratinization disorders. Ichthyosis is a group of genetically determined disorders that lead to excessive skin keratinization. ISO, thanks to its action in normalizing keratinization processes, can significantly improve the skin condition of patients with ichthyosis, especially in severe forms of the disease [66]. Research has shown that ISO effectively reduces hyperkeratosis and improves skin elasticity in patients with ichthyosis. Once the disease has been controlled with over 80% improvement, the general trend is to prescribe lower and increasingly spaced doses, allowing a “holiday” from oral retinoids during which the patient can be retinoid-free for 1–3 months. Minor recurrences are observed in severe keratinization disorders, such as lamellar ichthyosis (LI) and hyperkeratotic epidermolysis (EH) [67,68].

In some cases, retinol and ISO been used as part of adjuvant therapy in treating advanced forms of skin cancer [69,70]. They are most effective in cases of squamous cell carcinoma. ISO’s antineoplastic action may contribute to tumor size reduction and delay disease progression. In two clinical studies, ISO at doses of 1 mg/kg/day and 0.6 mg/kg/day, respectively, in combination with alpha-IFN (3 × 10^6^ I.U./day and 6 × 10^6^ I.U./day, respectively), achieved a total or partial therapeutic effect. However, the use of isotretinoin in oncology requires further research to accurately determine its efficacy and safety in this role.

It is also worth mentioning the possibility of using ISO in the treatment of Keratoacanthoma (KA), a highly differentiated variant of squamous cell carcinoma characterized by rapid growth over a course of several months. Histologically and clinically, it resembles squamous cell carcinoma and originates from hair follicle cells. There are case reports indicating the effective application of ISO in patients with KA, causing regression of the lesions after just two weeks of use, and even instances of complete cure [71,72].

In psoriasis, ISO exerts multiple effects on the skin that may potentially contribute to its regression. Isotretinoin can normalize the differentiation of keratinocytes, the predominant cells in the epidermis. Psoriasis is marked by an accelerated cycle of cell turnover, resulting in the buildup of scales. The capacity of isotretinoin to control the growth of keratinocytes could aid in diminishing the scaliness associated with psoriasis. There have been case reports and small clinical trials indicating that isotretinoin can induce remission in some patients with pustular psoriasis. In a specific randomized clinical study, a regimen combining narrowband ultraviolet B (NBUVB) with isotretinoin (0.5 mg/kg/day) was administered, whereas the control group was treated with NBUVB and a placebo. The group with ISO achieved a higher remission rate than the placebo group, although the result was not statistically significant. It was concluded that adding ISO to phototherapy shortens the duration of therapy and reduces the necessary radiation dose. There was an article published about five cases where traditional treatments (phototherapy and methotrexate) were ineffective, and the use of ISO achieved some clinical effect [73,74,75].

Itis difficult not to mention the positive effect that retinoids have in the case of mycosis fungoides and Sézary syndrome. Cutaneous T-cell lymphoma (CTCL) is a heterogeneous group of lymphoproliferative disorders of T lymphocytes, manifested by skin lesions. Mycosis fungoides presents numerous diagnostic challenges, leading to prolonged diagnostic processes. In its early stages, it can often be mistaken for psoriasis, lichen planus, or skin mycosis. In the publication found, it is noted that patients with early-stage mycosis fungoides may have a projected median survival of 10–35 years. However, more than 25% of patients may experience disease progression to an advanced stage, in which the median survival drops below four years, and in cases involving lymph nodes, it may only be 13 months. Sézary syndrome (a leukemic subtype of cutaneous T-cell lymphoma (CTCL) is characterized by the triad of erythroderma, lymphadenopathy, and the presence of Sézary cells in the skin, lymph nodes, and peripheral blood. Three traditional retinoids, isotretinoin, etretinate, and acitretin, acting via RAR, are the first retinoids tested for activity in CTCL. Isotretinoin is effective in the early and advanced stages of mycosis fungoides as an initial treatment to induce rapid remission. One of the retinoids offering hope for the treatment of mycosis fungoides and Sézary syndrome is bexarotene. In one publication, we can even read about a 44% drug response rate among 66 patients, where 9% of patients had a complete response to the drug, 35% had a partial response, and 23% experienced a stabilization of the course of the disease [76,77,78].

Acute myeloid leukemia (AML) is characterized by uncontrolled proliferation and impaired differentiation, leading to the accumulation of immature cells known as blasts. Despite advancements in AML treatment over the past 30 years, over 50% of young adults and 90% of older patients still succumb to the disease. The treatment of a specific AML subtype, acute promyelocytic leukemia (APL), has given rise to hopes that therapies based on ATRA may enhance outcomes in other AML subtypes. In APL, the fusion of the C-terminus of retinoic acid receptor α (RARα) on chromosome 17 with the N-terminus of promyelocytic leukemia protein (PML) on chromosome 15 is a common genetic alteration. This fusion results in the formation of the PML-RARα fusion protein, exerting a dominant negative effect on retinoic acid signaling. This inhibits differentiation by recruiting abnormal transcription factors and histone-modifying enzymes to crucial genes. At pharmacological concentrations, ATRA effectively binds to PML-RARα, overcoming its inhibitory effects and allowing the transcription of target genes. APL progenitors exposed to ATRA in vitro or during clinical treatment continue their differentiation program into neutrophils, ultimately undergoing senescence [79,80,81].

The precursor of RA, known as vitamin A or retinol, plays distinct roles in the development and maintenance of various cellular systems in mammals. Due to the detrimental effects of both RA deficiency and excess, some of which can be incompatible with life, organisms have evolved feedback mechanisms to regulate retinoid levels [79]. Consequently, the levels of RA in tissues reflect a delicate balance between biosynthesis from vitamin A and inactivation, primarily carried out by the cytochrome P450 (CYP) 26 family. While hepatic CYP26 plays a crucial role in preserving systemic retinoid homeostasis, recent findings suggest that these enzymes also contribute to the local control of RA signaling within specific microenvironments. In fetal gonads, for instance, the expression of CYP26B1 by Sertoli cells determines the fate of germ cells by modulating the bioavailability of ATRA [82,83,84].

ATRA have demonstrated significant anti-proliferative effects against chronic myelogenous leukemia (CML) cells in laboratory studies. Initial research involving CML patients with the Philadelphia chromosome indicated that ATRA could temporarily reduce the number of blast cells in the bone marrow or peripheral blood during certain disease phases. However, for this group of advanced CML patients who had received prior treatments, ATRA alone proved ineffective as a long-term therapy. The observed anti-leukemic effect in some patients points to the necessity for further research on retinoids in various treatment protocols and combinations for CML patients [85]. 

The list of diseases with isotretinoin/retinol treatment regimen and dosage with results according to the literature is presented in Table 2.

Table 3 presents an examination of issues concerning the safety and toxicity of retinoids, especially in the context of drug repurposing, with examples of side effects and possible hazards linked to retinoid-based treatments.

## 4. Conclusions

Vitamin A and its metabolite, retinoic acid (RA), play critical roles in various biological functions essential for the development and maintenance of organisms. These functions range from tissue differentiation, growth, and embryonic development to immune function and regulation of tumor suppressor genes. The diverse biological activities of retinoids, particularly all-trans retinoic acid (ATRA), have led to their exploration in drug repurposing strategies, which offer an efficient and cost-effective approach to discovering new therapeutic applications for existing drugs. The quest for potent retinoids with enhanced efficacy and reduced toxicity continues, with ongoing efforts focused on developing stable synthetic analogues that overcome the challenges associated with natural retinoids. Structure–activity relationships and modifications in retinoid structures aim to optimize their binding affinity and specificity to retinoid receptors, allowing for selective modulation of retinoid-related mechanisms. ATRA exhibits a diverse range of biological activities crucial in chordate embryogenesis, adult homeostasis, and various therapeutic applications. Clinical applications of retinoids, particularly isotretinoin (ISO), extend beyond acne treatment to various dermatological conditions such as rosacea, Hidradenitis suppurativa (HS), ichthyosis, and even certain forms of skin cancer. Additionally, ATRA shows promise in the treatment of diseases beyond dermatology, including mycosis fungoides, acute promyelocytic leukemia (APL), and chronic myelogenous leukemia (CML). The complex regulatory mechanisms governing retinoid levels and signaling pathways highlight the delicate balance between retinoid biosynthesis and inactivation, as well as the importance of feedback mechanisms in maintaining retinoid homeostasis. Further research is needed to fully understand the therapeutic potential of retinoids in various diseases and to develop effective treatment protocols and combinations for optimal patient outcomes. Potential benefits of drug repurposing with retinoids are significant, but several gaps in knowledge must be addressed to fully harness their therapeutic potential. Focusing on these areas can pave the way for new and improved treatments based on retinoid-based therapies. Retinoids are associated with certain toxicities and side effects, including teratogenicity and hepatotoxicity. A significant gap in knowledge exists regarding how to mitigate these risks while preserving therapeutic benefits. Research could focus on identifying safer retinoid derivatives or optimizing dosing strategies to minimize adverse effects. Innovative drug delivery systems could enhance the therapeutic potential of retinoids while reducing toxicity. Future research could explore alternative delivery methods, such as nanoparticles or liposomes, to improve bioavailability and target specific tissues or organs, thus, minimizing systemic side effects. Retinoids are well-established in dermatology, but their potential in other therapeutic areas, such as oncology, neurology, and immunology, is not fully understood. Research could explore the broader therapeutic applications of retinoids, focusing on how these compounds can be repurposed for other conditions. This includes evaluating their efficacy, safety profiles, and dosing requirements in different therapeutic contexts. Understanding the relationship between retinoid structures and their bioactivity is crucial. While significant progress has been made in characterizing basic retinoid structures, there is still much to learn about how subtle changes in chemical structure affect biological activity, toxicity, and therapeutic outcomes. Future research could focus on identifying the most effective retinoid structures for specific therapeutic targets.

## Figures and Tables

**Table 1:** Distinct differences in LBP of the RAR isotype, according to Ref. [31].

Helices	RARα	RARβ	RARγ
H3	Ser232	Arg225	Arg234
H5	Ile270	Ile263	Met272
H11	Val395	Val388	Ala397

**Table 2:** Disease units with treatment regimen of isotretinoins/retinoids and dosage with result according to references.

Disease Unit	Medication	Dosage	Results	Ref.
Acne Vulgaris	Isotretinoin	0.5–1.0 mg/kg/day; mean of 38.4 mg/kg cumulative dose	*n* = 305; remission: 87.64% (267), no effect: 12.46% (38)	[59]
Rosacea	Isotretinoin	0.5–1.0 mg/kg/day; mean of 33.3 mg/kg cumulative dose	*n*= 70 patients; full effect 34% (24), partial effect 57% (40)	[86]
Hidradenitis Suppurativa	Isotretinoin	0.45 ± 0.20 mg/kg/day (range: 0.14–0.95)	*n* = 25; 36% (9/25) complete responses; 32% (8/26) partial responses; 32% (8/25) no responses	[63]
Ichthyosis	Isotretinoin	1.83–2.05 mg/kg/day	*n* = 18; visible improvement: 60% (11/18)	[87]
SCC	IFNα–2a + Isotretinoin	mean of 1.0 mg/kg/day	*n* = 32; overall response rate: 68%, complete response rate: 25%	[70]
Keratoacanthoma	Isotretinoin	mean of 1.0 mg/kg/day	*n* = 1 CASE REPORT	[71]
Psorasis	narrow band ultraviolet B (NBUVB) + Isotretinoin	0.5 mg/kg/day	*n* = 17; 82% (*n* = 14) complete clearing of psorasis plaques	[73]
Mycosis Fungoides and Sézary syndrome	Bexarotene	150–300 mg/day	*n* = 66; 9% (*n* = 6) complete response, 35% (*n* = 23) partial response, 23% (*n* = 15) stabilized disease	[77]
Folliculitis Decalvans	Isotretinoin	0.1–1.02 mg/kg/day	*n* = 39; 82% full remission, 66% never relapsed	[88]
Dissecting Cellulitis	Isotretinoin	0.5–0.8 mg/kg/day	*n* = 51; 92% temporary remissions	[89]
Cutaneous Lupus Erythematosus	Isotretinoin	0.15–0.5 mg/kg/day	*n* = 24; 86.9% major clinical improvement or full clearing of lesions	[90]
Lichen Planus	Isotretinoin	20 mg/day	*n* = 27; 21.8% (*n* = 7) good response, 55.7% (*n* = 15), moderate improvement	[91]
Granuloma Annulare	Isotretinoin	40–80 mg/day (for 1 year)	*n* = 1 CASE REPORT	[92]
Leucoplakia	Isotretinoin	0.5 mg/kg/day	*n* = 53; 92% (*n* = 22) clinical response or stabilization of lesions	[93]
Darier’s Disease	Isotretinoin	0.5–4 mg/kg/day	*n* = 119 (metanalysis); 75–100% lesionclearance at 1st week, 80–100%lesion relapse within 7 days to 6 months post-treatment	[94]

**Table 3:** Adverse effects of retinoid therapy according to Ref. [95].

Topical	Adverse Effects
Skin	irritation, dryness, peeling, erythema and pruritus
**System**	**Adverse Effects**
Mucocutaneous	cheilitis, dryness of the oral mucosa, epistaxis, xerophthalmia, xerosis, fingertip fissuring, hair loss, nail fragility, periungual granuloma, paronychia
Musculoskeletal	myalgias, arthralgias, bony pain, premature fusion of the epiphyses, skeletal hyperostosis, calcification of tendons and ligaments
Neurologic	headaches, pseudotumor cerebri
Ophthalmologic	nyctalopia
Gastrointestinal/Metabolic	nausea, abdominal pain, diarrhea, elevation in liver function tests, elevation in serum triglycerides and cholesterol
Teratogenicity	abnormalities of the central nervous system, face, heart, and thymus
Psychiatric	depression, irritability/aggression, suicidality, sleep disturbances, mania, psychosis

## Data Availability

Data sharing is not applicable to this article.
